# Peripheral Nerve Blocks in Adolescents with Medically Refractory Migraine: A Retrospective Observational Study

**DOI:** 10.3390/children13070886

**Published:** 2026-06-30

**Authors:** Hamit Acer, Beste Kipcak Yuzbasi

**Affiliations:** 1 Department of Pediatric Neurology (HA), Denizli State Hospital, 20010 Denizli, Türkiye; 2 Department of Pediatric Neurology (BKY), Pamukkale University, 20160 Denizli, Türkiye; byuzbasi@pau.edu.tr

**Keywords:** adolescent, headache management, migraine, occipital nerve block, peripheral nerve block, refractory migraine

## Abstract

**Highlights:**

**What are the main findings?**

- Peripheral nerve blocks (GON/LON) may be associated with reductions in migraine frequency and pain severity in adolescents with refractory migraine.
- The procedure appeared to be generally well tolerated, and the observed adverse effects were mild and transient.

**What is the implication of the main finding?**

- Peripheral nerve blocks may be considered a potential therapeutic approach for adolescents who do not respond adequately to preventive pharmacological treatments.
- This approach may contribute to multidisciplinary migraine management strategies in pediatric neurology practice.

**Abstract:**

**Objective:** To evaluate the efficacy and safety of peripheral nerve blocks (PNB) in adolescents with medically refractory migraine. **Materials and Methods:** Twenty-eight adolescents with inadequate response to preventive therapy were included. Greater occipital nerve (GON) and/or lesser occipital nerve (LON) blocks were performed in three sessions at 8-week intervals. Pain severity was assessed using the Visual Analog Scale (VAS), and a structured questionnaire evaluated pain relief and functional outcomes. **Results:** A total of 84 procedures were performed. The results revealed that the mean monthly migraine frequency decreased from 14.4 to 4.7 days after the third session and the mean VAS score decreased from 7.7 to 3.3. Eighteen patients showed marked improvement, six had partial benefit, and four had no response. Questionnaire findings indicated improved pain control and patient-reported daily functioning. Adverse effects were mild and transient; no serious complications occurred. **Conclusions:** Peripheral nerve blocks, particularly GON and LON blockade, may be associated with reductions in headache frequency and severity in adolescents with refractory migraine and may contribute to improvements in patient-reported daily functioning. However, these findings should be supported by larger controlled studies.

## 1. Introduction

Migraine is a primary headache disorder that negatively impacts quality of life and disrupts social functioning. Migraine attacks frequently result in significant functional impairment, with approximately 50% of patients requiring bed rest [1]. Migraine is a common neurological condition worldwide; however, it has not been extensively studied in adolescents. It affects approximately 8% of individuals aged 17 years and younger, with prevalence increasing with age [2]. Pediatric migraine has substantial health and social consequences, with the most pronounced effects observed in school performance and physical health. Children and adolescents with migraine report reduced quality of life, increased absenteeism from school, and poorer academic performance compared to their migraine-free peers [3]. Many pediatric migraine patients experience difficulties with school attendance and concentration during lessons. High noise levels in the school environment can exacerbate headache frequency, and children may return home with headaches after school, creating communication problems within the family. Additionally, mood and anxiety disorders are more likely to occur in children with migraine, which can further impair functioning and reduce social interactions with peers, thereby worsening the overall impact on quality of life [4].

Therefore, effective treatment of pediatric migraine is highly desirable. Although numerous studies have examined migraine management in the adult population, research in pediatric patients remains limited due to the inherent challenges of conducting studies in this group. Consequently, medical treatments for pediatric migraine are often extrapolated from adult data [5]. Therapies used for adult migraine may lack pediatric approval, may be ineffective in children, or may have unknown effects on the developing nervous system [6]. Furthermore, long-term medication use during adolescence can present adherence challenges. For these reasons, nerve block procedures may represent an effective alternative treatment option.

Nerve blocks have been utilized for various headache disorders, and such injections can be performed relatively easily in a variety of settings using different pharmacological agents.

## 2. Materials and Methods

This retrospective observational study included patients who underwent peripheral nerve block procedures between October 2023 and April 2025 at the Pediatric Neurology Clinics of Pamukkale University and Denizli State Hospital as part of routine clinical practice. Ethical approval for the retrospective analysis and publication of the collected clinical data was obtained from the Pamukkale University Ethics Committee (approval date: 24 June 2025; decision number: 12; document number: E-60116787-020-712247). Written informed consent was obtained from the parents, and assent was obtained from the children. Twenty-eight adolescent patients aged 14–18 years were included in the study. Patients who had been diagnosed with migraine for more than 6 months, which could not be adequately controlled despite appropriate treatment, were classified as having refractory migraine. Migraine diagnosis was established according to the International Classification of Headache Disorders, 3rd edition (ICHD-3) criteria. Patients with medically refractory chronic migraine or status migrainosus were enrolled. Nerve block therapy was considered in patients who failed to respond to prophylactic treatment administered for longer than 3 months at appropriate doses and durations. Refractory migraine was defined as the absence of significant clinical improvement despite the use of at least two preventive therapies at appropriate doses and durations, persistence of attacks causing substantial impairment in patient-reported daily functioning, and inadequate pain control despite acute treatments. In addition, secondary causes of headache were excluded, and all patients were evaluated for medication overuse headache. 

For the injection a 30-gauge needle was used. The injection site was cleansed with alcohol, and the external occipital protuberance was palpated. In patients with Migraine, the target point for the greater occipital nerve (GON) block was located 2 cm lateral and 2 cm inferior to the occipital protuberance. Each injection site received 1 mL of lidocaine hydrochloride (Jetokain Simplex^®^, 20 mg/mL injection solution; ADEKA İlaç Sanayi ve Ticaret A.Ş., Samsun, Türkiye). All procedures were performed bilaterally, and the maximum total block dose was limited to 2 mL. All procedures were performed using anatomical landmarks without ultrasound guidance and with a 30-gauge needle. For the lesser occipital nerve (LON) block, the target point was located 5 cm lateral and 2 cm inferior to the occipital protuberance. The procedure consisted of bilateral injections of 1 mL of 1% lidocaine. The intervention was performed in three sessions at 8-week intervals. Patients remained under observation in the clinic for one hour after the procedure.

All adverse events were recorded.

Patients received either GON, LON, or combined GON and LON blocks in the same session. A cooling spray without ethyl chloride was used as local anesthesia. LON selection was performed in combination with GON particularly in patients with prolonged headache duration and in those who exhibited tenderness on palpation in the postauricular or posterior occipital region [7,8,9]. Pain intensity was evaluated using the Visual Analog Scale (VAS), and a structured questionnaire was administered to assess treatment effectiveness (Table 1). 

VAS scores and questionnaire data were collected before the first injection (baseline) and one week after the second and third treatment sessions, allowing assessment of both short-term and cumulative treatment response. Descriptive statistics were used to summarize patient characteristics and treatment outcomes. Continuous variables are presented as mean ± standard deviation or median (interquartile range), as appropriate. Changes in monthly migraine frequency and VAS scores across repeated measurements were analyzed using the Friedman test. When a significant overall difference was detected, post hoc pairwise comparisons were performed using the Wilcoxon signed-rank test with Bonferroni correction. Effect sizes were calculated using Kendall’s W for the Friedman test and the effect size coefficient (r = Z/√N) for Wilcoxon signed-rank tests. Categorical variables are presented as frequencies and percentages. Statistical analyses were performed using *SPSS* software (version 26; IBM Corp., Armonk, NY, USA).

## 3. Results

A total of 28 adolescent patients were included in the study, consisting of 26 female and two male patients, aged between 14 and 17 years (mean age: 15 years and 8 months). Only patients whose migraine attack frequency and duration could not be adequately controlled despite pharmacological treatment were enrolled. The duration of medication use before PNB blockade ranged from 6 to 26 months. Ten patients were diagnosed with migraine with aura, seventeen with migraine without aura, and one patient had a combined diagnosis of epilepsy and migraine.

All patients received prophylactic treatment prior to the procedure. The most frequently used prophylactic agents were topiramate, flunarizine, and magnesium. Eight patients were on monotherapy, while twenty were receiving dual therapy. The most common combinations were flunarizine plus magnesium (fourteen patients) and topiramate plus magnesium (six patients). The patients’ ongoing prophylactic treatments were maintained at the same dosages throughout the study period. Oral or intravenous paracetamol was administered for the management of acute pain attacks. No additional intervention or recommendation regarding lifestyle modifications was provided to patients in the PNB group.

All patients underwent brain magnetic resonance imaging (MRI) as part of neuroimaging evaluation. Twenty-seven patients had normal MRI findings. The patient with comorbid epilepsy demonstrated an encephalomalacia area in the left temporal region secondary to a previous cerebrovascular event. Electroencephalography (EEG) was performed on all patients; however, focal epileptiform discharges were only observed in the patient with epilepsy.

The procedures performed were either GON block alone or combined GON and LON block. A total of 84 procedures were administered to 28 patients, with 24 sessions involving GON block alone and 60 sessions involving combined GON and LON block.

**Figure 1:**
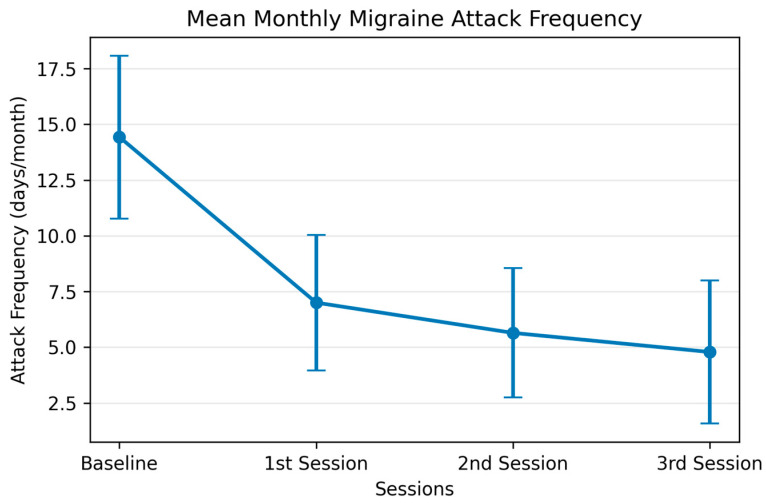
Reduction in mean monthly migraine attack frequency across treatment sessions.

Prior to intervention, mean attack frequency ranged from 10 to 20 days per month (mean: 14.4 days). Attack frequencies were reassessed in two-month intervals before each session. Following the first session, attack frequency decreased to between 2 and 12 days per month (mean: 7 days). After the second session, frequencies ranged from 2 to 11 days (mean: 5.6 days), and after the final session, from 1 to 10 days (mean: 4.7 days) (Figure 1).

The overall effect size for the repeated measures analysis was very large (Kendall’s W = 0.949). In addition, post hoc Wilcoxon signed-rank tests demonstrated large effect sizes for pairwise comparisons (*r* = 0.745–0.885), indicating a substantial treatment effect across repeated sessions. Four patients did not demonstrate a clinically meaningful reduction. Six patients experienced partial improvement, while eighteen patients demonstrated a marked reduction in attack frequency and daily performance improvement.

Adverse effects during the procedure included localized pain at the injection site, dizziness, which occurred in twelve sessions and minor bleeding, which occurred in two sessions. No serious or permanent adverse effects were observed. A cooling spray was used as local anesthesia in eighteen procedures, whereas no anesthesia was administered prior to the remaining sessions.

**Figure 2:**
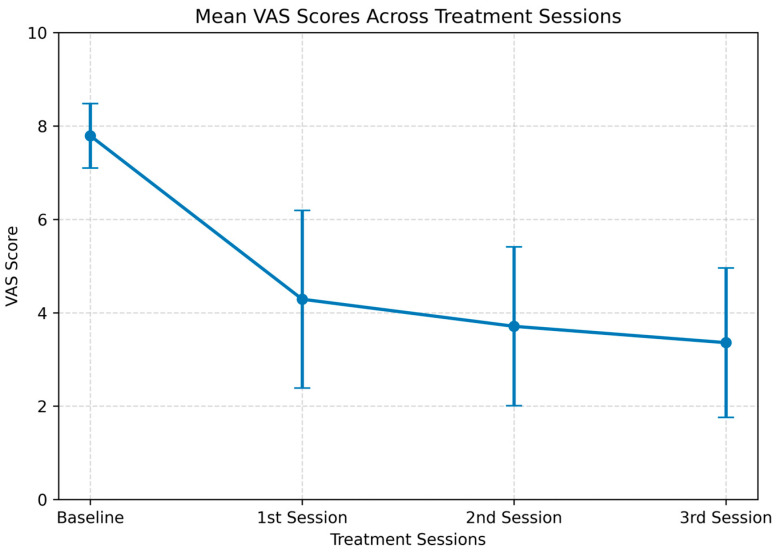
Change in mean VAS scores from baseline to after the third treatment session.

Pain severity was assessed using the VAS. Post hoc pairwise comparisons were performed using the Wilcoxon signed-rank test with Bonferroni correction (adjusted significance level *p* < 0.0083). Significant reductions in VAS scores were observed between baseline and all subsequent time points (all *p* < 0.001), as well as between the first and second sessions (*p* = 0.004) and the first and third sessions (*p* = 0.002). The difference between the second and third sessions did not reach statistical significance after correction (*p* = 0.012). Baseline VAS scores were 7.79 ± 0.69 (median: 8, IQR: 7–8). Mean VAS scores decreased to 4.29 ± 1.9 after the first session, 3.71 ± 1.7 after the second session, and 3.36 ± 1.6 after the third session. Median values also showed a consistent reduction over time [4 (3–5), 3 (2–5), and 3 (2–5), respectively]. (Figure 2).

The overall effect size for the repeated measures analysis was very large (Kendall’s W = 0.949). In addition, post hoc Wilcoxon signed-rank tests demonstrated large effect sizes for pairwise comparisons (*r* = 0.745–0.885), indicating a substantial treatment effect across repeated sessions.

All patients completed a questionnaire evaluating the effectiveness of the procedure. Questionnaire results are presented in Supplementary Table S1.

## 4. Discussion

This study suggests that greater and lesser occipital nerve blockade may be a safe and potentially effective intervention in adolescents with medically refractory migraine and may be associated with reductions in headache frequency and severity. Interventional migraine treatments are commonly used in adults; however, recent studies have demonstrated that they are also safe for use in pediatric and adolescent populations [10]. In a comprehensive survey study, 80% of 41 pediatric headache specialists reported regular use of peripheral nerve blocks (PNB), with 63% performing the procedure themselves [11]. Moreover, a recently published study by García Ron et al. evaluated the efficacy of GON blockade in pediatric patients presenting to the emergency department. These findings indicate that GON blockade can be used safely not only in scheduled outpatient settings but also in acute clinical situations [12]. In the adolescent population, long-term pharmacological therapy often poses challenges in terms of treatment adherence. Migraine can adversely affect academic performance, participation in group activities, interpersonal relationships, and the psychological well-being of the child [13]. Moreover, the presence of photophobia and phonophobia may render crowded classroom environments a potential trigger for migraine attacks. Therefore, effective management of migraine is crucial for preserving daily functional performance and ensuring continuity of academic life.

Among the most frequently targeted nerves are the GON, LON, auriculotemporal, supraorbital, and supratrochlear branches of the trigeminal nerve [14]. In our study, the targeted nerves were the GON and LON. The block consisted of local anesthetic, with or without additives such as corticosteroids. Corticosteroids have been associated with mixed results in migraine management and common adverse effects such as cutaneous atrophy when used in trigeminal nerve branches [15]. Therefore, lidocaine was used in our study because it is more acceptable in terms of the side effect profile. Potential adverse effects of PNB include minor complications such as vasovagal reactions, local infections, hypotension, and bleeding at the injection site, as well as more serious complications such as alopecia, anaphylaxis, hematoma, and nerve injury [16]. In our study, no major complications were observed; vasovagal reactions occurred in twelve procedures, bleeding at the injection site in two, and localized pain in twelve. Considering the safety profile, PNB was generally observed to be well tolerated in adolescents.

In our study, we evaluated the efficacy of GON blockade in adolescents with medically refractory migraine. Our findings suggest that GON blockade provides meaningful clinical improvement in both headache frequency and severity. The mean monthly headache frequency decreased from 14.4 days before treatment to 4.7 days after the third injection. These results are consistent with the efficacy rates reported in previous studies, such as 68% in the study by Son et al. and 62% in the study by Gelfand et al. [17,18]. The effectiveness was consistent with the results of other studies. While PNB is used for a variety of headaches, its greatest effectiveness is seen in migraine patients [19].

Pain severity was assessed using the VAS, a widely accepted and reliable method for headache evaluation [20,21]. The mean pre-procedure VAS score was 7.7, which decreased to 3.3 following the final injection, indicating the effectiveness of the intervention. All patients completed a post-procedure questionnaire designed based on validated assessment scales used in previous studies, focusing on daily living activities and treatment effectiveness [7]. The questionnaire included items evaluating daily functioning and procedural efficacy, with detailed individual responses presented in Supplementary Table S1. The intervention demonstrated a marked positive impact on pain, sleep quality, and social functioning. In 20 patients, the therapeutic effect persisted for five weeks or longer. Although the duration of benefit reported in the literature varies, the results were comparable to those of the study by Puledda et al., in which the efficacy duration ranged from 3 to 9 weeks [19]. Although randomized placebo-controlled studies have demonstrated the efficacy of GON blockade in adults with chronic migraine, studies in pediatric and adolescent populations remain limited [22]. The available pediatric literature largely consists of retrospective and open-label studies. Therefore, while a placebo effect cannot be entirely excluded, the observed clinical improvement should be interpreted with caution. Nonetheless, our findings suggest that peripheral nerve block may represent a potentially beneficial therapeutic option for adolescents with refractory migraine. 

GON blockade is a novel and relatively underutilized intervention in adolescents. In our study, favorable outcomes were achieved in terms of both efficacy and safety. Given its ease of application, it may serve as a valuable treatment option in medication-resistant patients. To the best of our knowledge, this is the first study related to this subject conducted in Türkiye.

## 5. Limitations

This study has several limitations, including the small sample size, lack of a control group, and single-center design. The retrospective nature of data collection and the absence of long-term follow-up beyond the third session may limit the generalizability of the findings. Furthermore, in the absence of a control group, the observed effects cannot be definitively distinguished from placebo effects, regression to the mean, or the natural variability of the disease. In addition, the choice of intervention (GON block alone or combined GON and LON block) was based on clinical judgment rather than randomization. Owing to the limited sample size and the unequal distribution of intervention types, adjustment for intervention type or meaningful subgroup analyses could not be performed. Moreover, outcome measures were based on VAS scores and a structured but non-validated questionnaire, without the use of standardized pediatric migraine disability scales. Larger, randomized controlled studies are needed to confirm these findings.

## 6. Conclusions

Greater and lesser occipital nerve blocks were well tolerated and were associated with reductions in headache frequency and pain severity in adolescents with medically refractory migraine. This minimally invasive intervention may reduce headache frequency and severity while improving patient-reported daily functioning. These findings support the consideration of occipital nerve block as a viable interventional option in adolescents who do not respond adequately to pharmacologic therapy.

## Figures and Tables

**Table 1:** Patient-reported outcome questionnaire.

Question	Answer Options
Did the intervention reduce your pain?	Yes, definitely; Yes, to some extent; No
If yes, how long did the effect last?	>8 weeks; 5–8 weeks; <4 weeks
Did the intervention improve your daily functioning?	Yes, definitely; Yes, to some extent; No
Would you have the procedure repeated?	Yes, definitely; Undecided; No
Overall, how satisfied are you with the outcome of the intervention?	Excellent; Good; Fair; Not satisfied
Have you experienced any side effects or complications?	Yes, significant side effects; Yes, mild side effects; Minimal side effects; No

## Data Availability

The data that support the findings of this study are available from the corresponding author upon reasonable request.

## Supplementary Materials

The following supporting information can be downloaded at: https://www.mdpi.com/article/10.3390/children13070886/s1, Table S1: Individual patient-reported outcome assessment.

## Abbreviations

EEG	electroencephalography
GON	greater occipital nerve
ICHD-3	International Classification of Headache Disorders, 3rd edition
LON	lesser occipital nerve
MRI	magnetic resonance imaging
PNB	peripheral nerve block
VAS	Visual Analog Scale
