# Supplementary material for: Peripheral Nerve Blocks in Adolescents with Medically Refractory Migraine: A Retrospective Observational Study
Source: Children (Basel). 2026 Jun 30;13(7):886. doi: 10.3390/children13070886 (PMC13406293; doi:10.3390/children13070886)
Supplement: Supplementary file 1 [file children-13-00886-s001.zip › children-4302845-supplementary.pdf]

Table S1. Individual Patient-Reported Outcome Assessment.

Patient ID	Did the intervention reduce your pain?	If yes, how long did the effect last?	Did the intervention improve your daily functioning?	Would you have the procedure repeated?	Overall, how satisfied are you with the outcome of the intervention?	Have you experienced any side effects or complications?
P-1	Yes, definitely	5–8 weeks	Yes, definitely	Yes, definitely	Excellent	Mild side effects
P-2	Yes, definitely	5–8 weeks	Yes, definitely	Yes, definitely	Excellent	No
P-3	Yes, definitely	>8 weeks	Yes, definitely	Yes, definitely	Excellent	No
P-4	No	<4 weeks	No	No	Not satisfied	Mild side effects
P-5	Yes, definitely	>8 weeks	Yes, definitely	Yes, definitely	Good	No
P-6	Yes, definitely	5–8 weeks	Yes, definitely	Yes, definitely	Good	No
P-7	Yes, definitely	>8 weeks	Yes, definitely	Yes, definitely	Excellent	Mild side effects
P-8	Yes, definitely	5–8 weeks	Yes, definitely	Yes, definitely	Excellent	Mild side effects
P-9	Yes, to some extent	<4 weeks	Yes, to some extent	Undecided	Fair	Mild side effects
P-10	No	<4 weeks	No	No	Not satisfied	No
P-11	Yes, to some extent	5–8 weeks	Yes, to some extent	Undecided	Fair	No
P-12	Yes, definitely	>8 weeks	Yes, definitely	Yes, definitely	Good	No
P-13	Yes, definitely	>8 weeks	Yes, definitely	Yes, definitely	Good	Mild side effects
P-14	No	<4 weeks	No	No	Not satisfied	No
P-15	Yes, to some extent	<4 weeks	Yes, to some extent	Undecided	Fair	No
P-16	Yes, definitely	5–8 weeks	Yes, definitely	Yes, definitely	Excellent	Mild side effects
P-17	Yes, definitely	5–8 weeks	Yes, definitely	Yes, definitely	Excellent	No
P-18	Yes, definitely	>8 weeks	Yes, definitely	Yes, definitely	Excellent	No
P-19	Yes, definitely	5–8 weeks	Yes, definitely	Yes, definitely	Good	No
P-20	Yes, definitely	>8 weeks	Yes, definitely	Yes, definitely	Excellent	Mild side effects
P-21	Yes, definitely	5–8 weeks	Yes, definitely	Yes, definitely	Excellent	Mild side effects
P-22	Yes, definitely	>8 weeks	Yes, definitely	Yes, definitely	Good	No
P-23	Yes, definitely	>8 weeks	Yes, definitely	Yes, definitely	Good	Mild side effects
P-24	No	<4 weeks	No	No	Not satisfied	No
P-25	Yes, to some extent	<4 weeks	Yes, to some extent	Undecided	Fair	No
P-26	No	<4 weeks	No	No	Not satisfied	Mild side effects
P-27	Yes, definitely	>8 weeks	Yes, definitely	Yes, definitely	Good	No
P-28	Yes, definitely	5–8 weeks	Yes, definitely	Yes, definitely	Good	No
